# Plasminogen Alleles Influence Susceptibility to Invasive Aspergillosis

**DOI:** 10.1371/journal.pgen.1000101

**Published:** 2008-06-20

**Authors:** Aimee K. Zaas, Guochun Liao, Jason W. Chien, Clarice Weinberg, David Shore, Steven S. Giles, Kieren A. Marr, Jonathan Usuka, Lauranell H. Burch, Lalith Perera, John R. Perfect, Gary Peltz, David A. Schwartz

**Affiliations:** 1 Duke University Medical Center, Durham, North Carolina, United States of America; 2 Roche Palo Alto, Palo Alto, California, United States of America; 3 Fred Hutchinson Cancer Research Center, Seattle, Washington, United States of America; 4 National Institute of Environmental Health Sciences, Research Triangle Park, North Carolina, United States of America; 5 University of Wisconsin, Madison, Wisconsin, United States of America; 6 Oregon Health and Science University, Portland, Oregon, United States of America; The Wellcome Trust Centre for Human Genetics, University of Oxford, United Kingdom

## Abstract

Invasive aspergillosis (IA) is a common and life-threatening infection in immunocompromised individuals. A number of environmental and epidemiologic risk factors for developing IA have been identified. However, genetic factors that affect risk for developing IA have not been clearly identified. We report that host genetic differences influence outcome following establishment of pulmonary aspergillosis in an exogenously immune suppressed mouse model. Computational haplotype-based genetic analysis indicated that genetic variation within the biologically plausible positional candidate gene plasminogen (*Plg*; Gene ID 18855) correlated with murine outcome. There was a single nonsynonymous coding change (*Gly110Ser*) where the minor allele was found in all of the susceptible strains, but not in the resistant strains. A nonsynonymous single nucleotide polymorphism (*Asp472Asn*) was also identified in the human homolog (*PLG*; Gene ID 5340). An association study within a cohort of 236 allogeneic hematopoietic stem cell transplant (HSCT) recipients revealed that alleles at this SNP significantly affected the risk of developing IA after HSCT. Furthermore, we demonstrated that plasminogen directly binds to *Aspergillus fumigatus*. We propose that genetic variation within the plasminogen pathway influences the pathogenesis of this invasive fungal infection.

## Author Summary

Invasive aspergillosis (IA) is the most common invasive mould infection among highly immune compromised hosts. While exogenous immune suppression is the greatest risk factor for infection acquisition, polymorphic variation in key immune effector genes likely contributes to disease susceptibility as well. We hypothesized that susceptibility to invasive aspergillosis was, in part, due to host genetic variation. By screening strains of inbred mice, we demonstrated differential susceptibility to invasive aspergillosis. Using the Roche Mouse single nucleotide polymorphism (SNP) Database, we localized an area on murine chromosome 17 as a putative “quantitative trait locus” governing disease susceptibility. Within this interval, the gene encoding plasminogen (*Plg*) was found to have a coding change SNP in murine strains that were highly susceptible to disease. Many micro-organisms are recognized to hijack the plasminogen to be more prevalent in human hematopoietic stem cell transplant recipients who developed IA as compared to those who did not. Thus, we demonstrated a genetic basis for increased susceptibility to IA in both a murine model and human cohort, and implicated the fibrinolytic system in the pathogenesis of this disease.

## Introduction

Invasive infection with *Aspergillus fumigatus* (AF) is a common and life-threatening infection among severely immunocompromised individuals. Despite aggressive surveillance and prophylaxis, the incidence of invasive aspergillosis (IA) in hematopoietic stem cell transplant (HSCT) recipients remains approximately 10%, and the three month mortality following infection approaches 30% [1],[2]. AF infection leads to a potentially hemorrhagic bronchopneumonia, with angio-invasion and tissue destruction. Several factors have been shown to affect risk for developing IA in allogeneic HSCT recipients. These include immunosuppresion required for HSCT, graft-vs-host-disease, and corticosteroid use[2]. However, despite having similar risk profiles, only a subset of at-risk individuals will develop IA. Since most of the identified risk factors for IA affect the immune system of the recipient, we hypothesize that genetic variation within key innate or adaptive immune response genes could influence susceptibility to or outcome of this invasive fungal infection.

The susceptibility of different inbred murine strains for developing IA after immunosuppression was analyzed to determine if genetic factors affect susceptibility to aspergillosis. Haplotype-based computational genetic analysis of this survival data identified *PLG* as a candidate susceptibility gene. A SNP that caused a significant amino acid substitution in human *PLG* was identified, and human *PLG* alleles were found to affect risk for developing IA in HSCT recipients.

## Results

### Inbred Mouse Strains Have Distinct Survival Patterns after Invasive Pulmonary Aspergillosis

Ten inbred mouse strains were transiently immunosuppressed with cyclophosphamide and cortisone acetate prior to exposure to AF conidia. The strain-specific survival patterns were then evaluated over a 14 day period. This immunosuppressive regimen disrupts macrophage and neutrophil-mediated defense against inhaled conidia. In control experiments, no significant mortality was observed in the nine different immunosuppressed strains of mice that were not exposed to AF. However, the immunosuppressive regimen caused significant mortality in the DBA/2 strain. Therefore, subsequent analyses were performed with and without the data from this strain. Since the immunosuppressive regimen was initiated 3 days prior, all strains were rendered neutropenic at time of exposure to AF. The neutropenia extended through day 7 with neutrophil recovery by day 10 (Table S1). Quantitative PCR (qPCR) measurements performed on lung tissue harvested 24 hours after AF exposure indicated that the inbred strains were exposed to equivalent amounts of AF and had an equivalent pulmonary burden of *A. fumigatus* (Table S1). Thus, the inbred strains received the same immunosuppressive regimen, experienced identical periods of neutropenia, and received the same pulmonary fungal inoculum.

**Figure 1:**
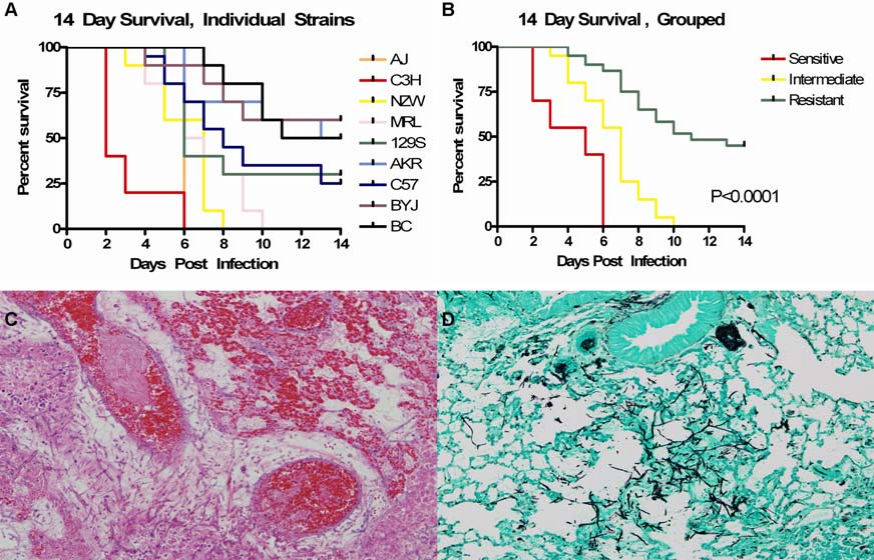
Survival following immune suppression and inhalation of *A. fumigatus* differs among inbred strains of mice. (A) 14-day survival phenotype for inbred murine strains following immunosuppression and inhalation of 3.0×10^8^ AF293 conidia. Susceptible strains had death of all mice by day 6 following infection. Intermediate strains had death predominately during neutrophil recovery (days 8–11) and resistant strains had surviving mice at day 14. N = 10 mice per strain. Repetitions of the inhalation in C57Bl/6, Balb/C, A/J and C3H/HeJ yielded similar results, indicating reproducibility of phenotype and consistency of inhalational protocol. (B) Kaplan-Meier curve representing divergent outcomes of murine strains following immune suppression and inhalation of *A. fumigatus*. Death occurred earlier in susceptible mice as compared to intermediate and resistant mice, as well as in intermediate mice as compared to resistant mice (p<0.001, log rank). (C) Haematoxylin and eosin stain of A/J lung, taken from a moribund mouse 4 days following immune suppression and inhalation of 3.0×10e8 AF conidia, magnification 40×. 1d. Gomori-methanamine silver stain of A/J lung, showing a focus of AF hyphae, magnification 40×.

Despite this, the inbred strains exhibited significant and reproducible differences in survival after exposure to AF (Figure 1A). The “susceptible” inbred strains (A/J and C3H/HeJ) exhibited 100% mortality by day 6. The DBA/2J strain also experienced 100% mortality by day 6; however the survival curves for immune suppressed unexposed DBA/2J mice were not significantly different from the mice that were exposed to AF (data not shown). The “intermediate” strains (MRL/MPJ and NZW/LacJ) also experienced 100% mortality; however the majority of deaths in these strains occurred between days 5 and 10. Five strains exhibited a “resistant” pattern of survival: (AKR/J, C57/Bl6J, 129/SvJ, Balb/CJ, and Balb/CByJ); they had between 30–60% survival at the end of the 14 day observation period (Figure 1B). The survival patterns were reproducible on repeated experimental trials. Histological evaluation of the lungs of moribund mice revealed *Aspergillus* hyphae and foci of pneumonitis (Figure 1C, D). Overt histologic differences between moribund mice from each strain were not noted, however histologic sections from surviving mice noted resolving pneumonia (data not shown). The amount of AF in the lung 48 hours after infection was also measured by qPCR. At 48 hours, the fungal burden in the sensitive strains (median conidial equivalents 4.7 log cells/gram lung tissue; range 3.8–5.0) was significantly greater (p = 0.02) than in the resistant strains (median conidial equivalents 3.7 log cells/gram lung tissue; range 2.7–5.3) (Table S1). Despite having the same level of immunosuppression and initial exposure to AF, the inbred strains exhibited significant differences in fungal burden and survival after AF exposure. These findings suggest that genetic factors affect the control of fungal burden, and survival in immune suppressed mice after AF exposure. Notably, C3H/HeJ mice are known to have defective TLR-4 signalling [3]. To evaluate the role of TLR-4 hypofunction in outcome following inhalation of *A. fumigatus*, both non-immune compromised and exogenously immune compromised C57Bl6^tlr4−/−^ mice were evaluated. These mice exhibited a “resistant” phenotype upon exposure to *A. fumigatus* in this model (data not shown). Thus, the survival pattern exhibited by C3H/HeJ mice in our model was not felt to be solely due to hypofunction of TLR4.

### Computational Genetic Analysis Identifies Plasminogen (*PLG*) as a Candidate Susceptibility Gene for Invasive Aspergillosis

**Figure 2:**
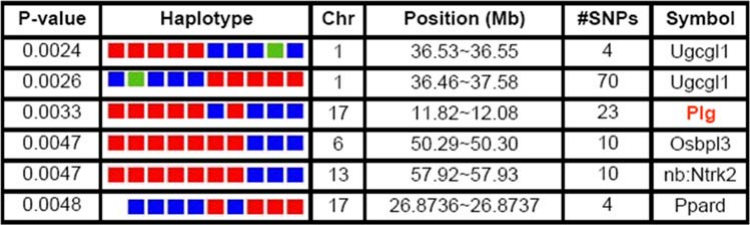
Computational genetic analysis identifies plasminogen (*PLG*) as a candidate susceptibility gene for invasive aspergillosis. Haplotype-based computational genetic analysis was used to analyze the area under the survival curve to identify genetic factors affecting survival after AF exposure among the inbred strains. Colored blocks represent haplotype structure, with red blocks representing “resistant” murine strains (C57, 129, Balb/C, Balb/CBy and AKR) and the “intermediate” strain NZW/LacJ and blue blocks representing “susceptible” murine strains (A/J, DBA/2J and C3H/HeJ) and the “intermediate” strain MR/MpJ. The green block represents missing haplotype data.

To identify genetic factors affecting survival after AF exposure, the inbred strain survival data were analyzed using haplotype-based computational genetic analysis [4],[5],[6]. This analysis identifies genomic regions where the pattern of genetic variation among inbred murine strains correlates with a pattern of phenotypic responses. The area under the survival curve (AUC) after AF exposure was analyzed; and 2 haplotype blocks were identified where the pattern of genetic variation had a strong correlation with survival (Figure 2, Figure S1A). The top two predicted loci contained the genes UDP-glucose ceramide glucosyltransferase-like 1 (*Ugcgl1*) and Plasminogen (*PLG*). While *Ugcgl1*, a glycoprotein glucosyltransferase, may have some role in CD4/CD8 thymocyte function [7], recent reports linking the fibrinolytic system with host response to infectious pathogens made *PLG* an attractive candidate for influencing susceptibility to IA [8]–[18]. Because the immunosuppressive regimen induced mortality in the DBA/2 strain, the computational analysis was also repeated using survival data that did not include this strain. When this reduced dataset was analyzed, *PLG* was among the genomic regions whose pattern of genetic variation correlated survival (Figure S1B, Figure S2).

*PLG* was sequenced across 20 inbred strains, and 423 SNPs were identified. Among the 10 inbred *Mus musculus* strains characterized here, the pattern of genetic variation in *PLG* was organized into 2 haplotype blocks. The 129, AKR, Balb/c, Balb/cBy, C57, and NZW strains had one haplotype, while the AJ, MRL, DBA and C3H strains had the other haplotype. There was a single non-synonymous SNP that altered an amino acid (G110S). This non-conservative amino acid substitution is within the first kringle domain, which regulates the initial binding of plasminogen to fibrin as well as plasmin-induced cell-detachment [19],[20]. The glycine at this position is conserved across all five kringle domains of plasminogen in mice and humans, suggesting that this amino acid change may have significant functional importance. Notably, the susceptible strains had the minor allele (Ser) at this position, which was not present in the resistant strains.

### Human *PLG* Alleles also Affect Susceptibility to Invasive Aspergillosis

Since an allelic difference in murine *PLG* was associated with susceptibility in a mouse model, it was possible that polymorphisms in human plasminogen (*PLG*) could also affect susceptibility to IA. If true, the best chance for detecting a genetic effect would be through analysis of a cohort of immunosuppressed patients that is at increased risk of developing invasive aspergillosis. The incidence of invasive aspergillosis in allogeneic HSCT recipients, which undergo intensive immunosuppression is approximately 10% [1],[2]. Therefore, HSCT recipients, followed for at least one year after transplant, provide an ideal population for identifying the genetic factors associated with IA susceptibility (Table 1).

**Table 1:** Demographics of HSCT Recipients.

	IA +			IA −		P value
	(n = 83)			(n = 147)		
Characteristic		no	%	no	%	
**Age (yr)**						
**Gender**						
	Male	48	58%	70	48%	NS
	Female	35	42%	77	52%	
**Transplant Type**						
	MatchRelated	26	31%	11	7%	0.01
	MatchUnrelated	53	64%	135	92%	
	Mismatch	4	5%	1	1%	
**Malignancy**						
	CML	37	45%	68	46%	NS
	CLL	1	1%	0	0%	NS
	ALL	7	8%	23	16%	0.04
	ANL	16	19%	26	18%	NS
	NHL	1	1%	0	0%	NS
	AA	2	2%	2	1%	NS
	MDS	10	12%	26	18%	0.01
	MM	7	8%	1	1%	NS
	other	2	2%	1	1%	NS
**Stem Cell Source**						
	Bone Marrow	79	95%	130	88%	NS
	PBSC	4	5%	17	12%	
**AGVHD**						
	None	10	12%	14	10%	NS (none vs any GVHD)
	Mild	32	39%	90	61%	0.001 (none/mild vs severe)
	Severe	41	49%	43	29%	
**CGVHD**						
	None/Mild	3	7%	10	9%	NS
	Mild	12	27%	18	17%	
	Severe	30	67%	81	75%	
	Censored*	34		6		
	Missing	4		21		
**Allele Frequency**						
**A**			33%		23%	
**G**			66%		76%	

*: subjects were censored if follow-up ended prior to day 100% calculated based on total persons in each time period IA = invasive aspergillosis CLL = chronic lymphocytic leukemia; CML = chronic myelocytic leukemia; ALL = acute lymphoblastic leukemia AML = acute myeloblastic leukemia; NHL = non-Hodgkin's lymphoma; PBSC = peripheral blood stem cells ANL = acute nonlymphoblastic leukemia; AA = aplastic anemia; MM = multiple myeloma; MDS = myelodysplastic syndrome; AGVHD = acute graft-versus-host disease; CGVHD = chronic graft-versus-host disease.

Of 87 total Caucasian HSCT donor-recipient pairs with IA sequenced, two were excluded due to missing data and two were excluded due to IA occurring during a second stem cell transplant. Only 2 of a total of 149 HSCT donor-recipient pairs that did not develop IA were excluded from this analysis. One pair was excluded due to having a second stem cell transplant and one was excluded due to missing data.

To perform the human genetic study, we first had to characterize the pattern of genetic variation in the human plasminogen gene. To do this, all exons and the promoter region of *PLG* were sequenced in 20 HSCT donor-recipient pairs (40 DNA samples from 20 stem cell donors and 20 stem cell recipients). Although 4 SNPs causing an amino acid change were identified, only one of these SNPs had a minor allele frequency above 1%. Therefore, our analysis focused upon SNP rs4252125 (Asp472Asn), which had a minor allele frequency (Asn472) of 25%. This non-conservative (neutral to acidic amino acid) substitution occurs in a loop region that connects the 4th and 5^th^ kringle domains of plasminogen. This loop region can form highly variable structures based on the surrounding environment. Similar to the murine polymorphism, this amino acid change could have a significant functional impact through altering the alignment of kringle domains or ligand binding.

**Figure 3:**
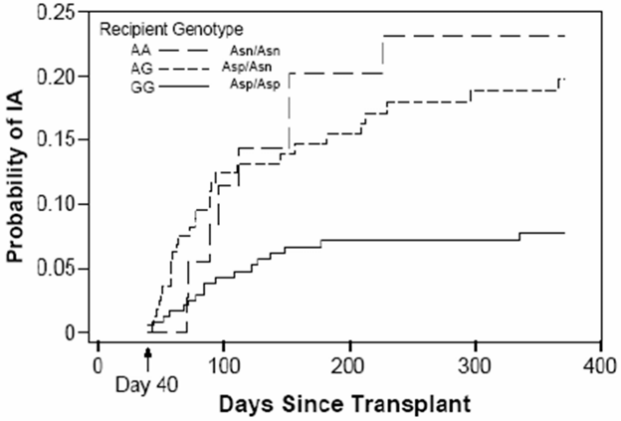
Kaplan-Meier curve demonstrating elevated risk of developing IA in HSCT recipients with AA or AG genotype, using GG as the reference genotype. The data represent 59 HSCT recipients who developed IA between days 40–365 following transplantation and 135 HSCT recipients who survived >40 days following HSCT and did not develop IA (HR genotype AA 5.56, 95% CI 1.88, 16.48; HR genotype AG 3.00 95% CI 1.46, 6.14; p<0.0005). The genotypic results are reported for follow-up beyond 40 days, using a left-censored analysis.

The genotype at Asp472Asn for the remainder of the HSCT cohort was then determined. Genotype was interpreted in a blinded manner, such that the two independent persons analyzing genotype data were unaware of each subject's status as a case or control. Analysis of these data indicated that HSCT recipients that were homozygous or heterozygous for Asn472 (AA or AG genotype) were at a significantly increased risk of developing IA after transplant. The Asn472 allele was present with greater frequency in HSCT recipients who developed IA (47/83; 56%) as compared to those who did not (62/147; 42%). Importantly, the genotype-specific risk for developing IA was not constant over time after transplant. Risk for development of IA following HSCT is bimodal, with peak risk periods occurring during the pre-engraftment phase (<day 40) and a second peak during the post-engraftment phase, during days 40–100 [2]. To allow for possible violations of the proportional hazards assumption, the genotypic hazard ratios were evaluated during 3 distinct time periods post HSCT: days 0–40, days 40–100 and days 100–365. There was a constant and substantial elevation in the genotype-specific hazard ratio for IA among recipients who carried at least one “Asn” allele (AA or AG genotype) between 40–365 days following HSCT (Figure 3, Table 2). Perhaps due to a low number of cases of IA occurring during days 0–40 or to the confounding effects of known strong risk factors pre-engraftment (e.g. neutropenia), an association between *PLG* genotype and IA was not observed during the first 40 days after transplant. For follow-up after day 40, the recipient plasminogen genotype remained a significant risk factor for IA susceptibility (p<0.0005) when a multivariate analysis was used to simultaneously account for effects of other factors that were significant in univariate analysis (HLA matching status, source of stem cells, and underlying malignancy). In addition, there was an apparent gene-dosage effect: homozygous AsnAsn individuals were at a 5.6-fold increased risk of developing IA, while heterozygous individuals had a 3.0 fold increased risk, relative to AspAsp individuals. A majority (96%) of subjects received fluconazole prophylaxis (no anti-*Aspergillus* activity), with the remainder receiving itraconazole, which does possess anti-*Aspergillus* activity (4%). Type of prophylaxis did not differ significantly between cases and controls. Inclusion of prophylaxis in the risk model revealed that the type of prophylaxis was not predictive of acquiring invasive aspergillosis, nor did the results related to genotype change with adjustment for type of prophylaxis. Notably, donor polymorphism status was not associated with development of aspergillosis. This served as an important negative control; plasminogen is synthesized hepatically, and thus, the donor plasminogen genotype should not associate with disease acquisition.

**Table 2:** Recipient Plg genotype is a risk factor for development of invasive aspergillosis after day 40 following HSCT.

Genotype:	Adjusted* Hazard Ratio	95% Confidence Interval
**AA (Asn/Asn)**	5.56	(1.88, 16.48)
**AG (Asp/Asn)**	3.00	(1.46, 6.14)
GG (Asp/Asp) is referent category *Adjusted for cell type, HLA matching, and malignancy category. p<0.0005		

HSCT recipients with “AA” (Asn/Asn) or “AG” (Asp/Asn) genotype are at increased risk of developing invasive aspergillosis following transplant as compared to HSCT recipients with “GG” (Asp/Asp) genotype. The data were adjusted for cell type, HLA match, and malignancy category. The “GG” (Asp/Asp) genotype is the referent category.

During the 40–365 days following transplant, 59 HSCT recipients developed IA; and 135 HSCT recipients who survived for more than 40 days following HSCT did not develop IA. The analysis of survival following diagnosis of IA showed somewhat reduced risk of death among IA patients with the same genotypes, with the hazard ratio for AsnAsn = 0.32 95% CI = (0.11, 0.89) and the hazard ratio for AspAsn = 0.79, 95% CI = (0.43, 1.44) (Table 2). Thus, an allelic difference causing an amino acid change in plasminogen is associated with increased risk for developing IA in a high-risk human cohort, as well as in an experimental murine model, and may also be associated with survival in HSCT patients who develop IA (p = 0.07).

### Plasminogen Interacts with AF

**Figure 4:**
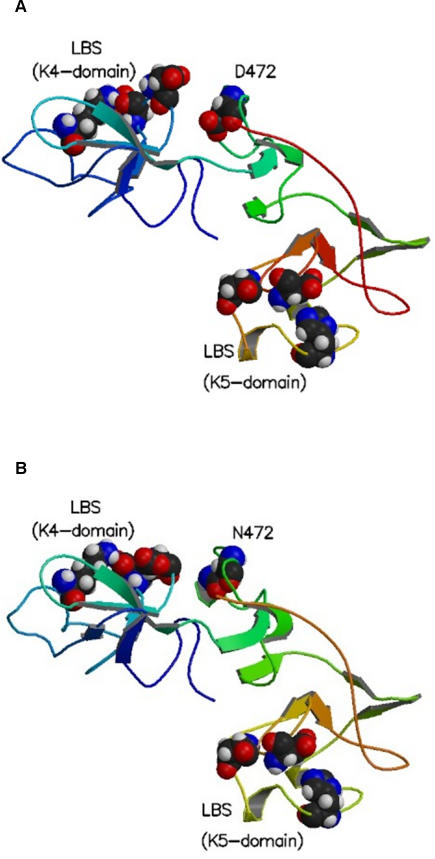
Ribbon diagrams of the combined kringle 4 (K4) and kringle 5 (K5) domains of human plasminogen. (A) Ribbon diagram showing Asp472 and the relationship to a major lysine binding site (LBS) on kringle 4. (B) Asp472 in the native K4K5 structure mutated to Asn.

**Figure 5:**
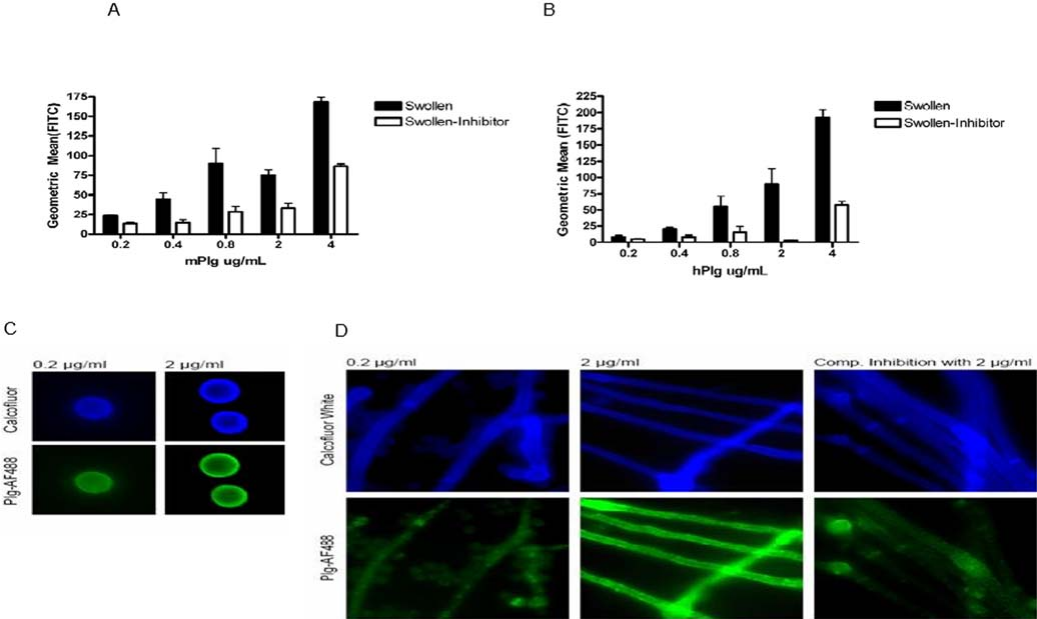
Murine and human plasminogen bind swollen *A. fumigatus* conidia and hyphae. (A and B) *A. fumigatus* conidia (1×10^6^) were incubated with several concentrations of murine (A) or human (B) *PLG*-AF488 (FITC) for 30 minutes at 37°C. Binding was assessed by flow cytometry. There was a strong correlation between the geometric mean of the FITC channel and *PLG*-AF488 concentration. Results are reported as the geometric mean±the standard error of the mean of at least three experiments. Specificity of binding is demonstrated as pre-incubation with 4 ug/mL unlabeled plasminogen for 30 minutes at 37°C inhibits binding of AF488-labeled plasminogen. Results are reported as the geometric mean±the standard error of three replicates. (C.) *A. fumigatus* conidia (1×10^6^) were stained with murine *PLG*-AF488 (0.2 µg/ml) and Calcoflour White for 30 minutes at 37°C. Plasminogen binding is evident on the conidial surface. (D.) *A. fumigatus* hyphae were incubated with several concentrations of murine *PLG*-AF488 for 30 minutes at 37°C and calcofluor white and visualized with fluorescence microscopy. Preincubation (30 minutes at 37°C) of hyphae with unlabelled plasminogen inhibits binding of AF488-labeled plasminogen.

We next sought to identify possible mechanisms by which plasminogen may influence susceptibility to invasive aspergillosis. The lysine binding sites in the 1^st^ and 4^th^ kringle domains of plasminogen mediate plasminogen binding to fibrinogen and other targets [20]. Analysis of the theoretical crystal structure of the murine kringle domain 1 revealed that substitution of serine for glycine at this position increased the negativity of the electrostatic potential around the principal lysine binding site. Such a change could increase the affinity of lysine binding to the 1^st^ kringle domain (Figure S3). Since plasminogen is more easily activated after binding, this amino acid substitution could increase the rate of plasminogen activation [12],[21],[22]. Analysis of the crystal structure of human plasminogen (Figure 4) kringle 4 (K4) and kringle 5 (K5) revealed that a substitution of asparagine for aspartic acid at position 472, located in the loop connecting K4 and K5, could potentially influence K4–K5 interactions or binding at the K4 lysine binding site (LBS). Although an interaction between plasminogen and AF has not been previously described, plasminogen does bind to other fungal pathogens [14],[23]. Therefore, immunofluorescence microscopy and flow cytometric analysis were used to determine if plasminogen bound to AF. Immunofluorescence microscopy demonstrated that FITC-labeled murine (Figure 5C, D) bound to both swollen AF conidia and hyphae in a dose dependent manner. Neither murine nor human plasminogen bound resting conidia (data not shown). The dose-dependent binding of plasminogen to AF was confirmed and further quantified by flow cytometry (Figure 5A). Furthermore, specificity of the interaction was tested using pre-incubation of swollen conidia with unlabeled plasminogen. This pre-incubation inhibited the binding of the labeled plasminogen (Figure 5A). Human plasminogen bound to swollen conidia and hyphae (Figure 5B), and demonstrated similar specificity as murine plasminogen, with inhibition of binding by pre-incubation with unlabeled human plasminogen (Figure 5B). FITC-labeled bovine serum albumin (BSA) bound to AF conidia to a lesser degree than labeled plasminogen, and pre-incubation of AF conidia with 0.1% BSA did not inhibit binding of labeled plasminogen (data not shown), highlighting the specificity of the plasminogen-AF interaction. These observations indicate that both infectious forms of AF can directly bind plasminogen, which supports the possibility that the plasminogen system plays an important role in the pathogenesis of IA. This also suggests a plausible mechanism by which the polymorphisms could alter susceptibility to invasive aspergillosis. A polymorphism that enhances plasminogen binding to the pathogen will increase plasminogen activation on the pathogen surface, which could facilitate pathogen entry and pathogen-induced tissue damage [11].

## Discussion

Our genetic analyses in mice and humans indicate that plasminogen alleles affect risk for developing invasive aspergillosis in the immunocompromised state. This association is also supported by functional in vitro studies demonstrating that plasminogen binds to AF. To our knowledge, this is the first attempt to identify genetic polymorphisms affecting risk of IA using a multi-species genetic mapping approach, with a murine model system targeting a polymorphic susceptibility gene which is then assessed in a well-defined human cohort. This approach, unlike a pure candidate gene approach, allowed evaluation of a gene that would be unlikely to be selected a priori for evaluation. While clinical applications of this finding will require further prospective evaluation, the finding can serve as a proof-of-principle paradigm for this staged approach to identifying host genetic polymorphisms that can affect outcome in immune compromised patients. Identification of a genetic polymorphism that influences infectious outcome after HSCT has important implications for pre and post transplant care, and may also have implications for the management of other immune-compromised patients. For example, genetic testing could identify high risk individuals who may benefit from use of broad-spectrum antifungal agents or enhanced monitoring for infection. While plasminogen polymorphisms are associated with development of invasive aspergillosis in HSCT recipients, it remains to be determined whether this allelic variant affects the risk of developing invasive aspergillosis after chronic corticosteroid use, semi-invasive aspergillosis or allergic bronchopulmonary aspergillosis.

Additionally, these findings implicate the fibrinolytic pathway as an important mediator of fungal infection. The fibrinolytic pathway is increasingly recognized in infectious disease pathogenesis, and this is the first report to link plasminogen with a filamentous fungus. Several mechanisms may explain how genetic changes affecting the function of plasminogen and the fibrinolytic system could influence host susceptibility to IA. IA is characterized by hemorrhage and tissue destruction, which are mediated by the plasminogen system. As previously demonstrated for streptococcal infection [18], AF-induced plasminogen activation could trigger plasminogen-mediated destruction of extracellular matrix components, which in turn enhances tissue invasiveness of the pathogen. Genetic differences that alter the coagulative function of plasminogen may also influence disease pathogenesis by promoting pulmonary hemorrhage and infarction. Immobilization of plasminogen on the pathogen surface allows for easier activation of this zymogen[10]. This brings the activated enzyme into close contact with key substrates in the basement membrane, which increases the virulence of bacteria [9],[16],[24] and other fungi [13],[14],[23]. Since AF adheres to components in the extracellular matrix and basement membrane [25],[26], its ability to bind plasminogen could be an important virulence mechanism. Since plasminogen plays an important role is inflammation and host defense, this provides another potential mechanism for the plasminogen system to affect the pathogenesis of IA. Plasminogen is a direct chemotaxin and is an activator of monocytes, and has recently been shown to prevent monocyte apoptosis [27]–[30]. A bidirectional relationship between coagulation and inflammation has long been recognized [15],[22]. Since monocytes/macrophages are critical for host defense, genetic variation in plasminogen could cause subtle differences in immune function that may also affect outcome after exposure to AF in severely immune compromised hosts.

Although death rates following infection tended to be reduced among carriers of the same genotypes that conferred increased risk, this finding may well be due to chance, as it was not statistically significant (p = 0.07). However, puzzling the finding, if this proves statistically significant in another population, it is of profound importance since it dissociates risk from acquisition of disease from survival. Clinical outcome following development of IA hinges not only on immune recovery to limit fungal related pulmonary hemorrhage and tissue infarction, but also on control of an over-exuberant inflammatory response as the immune system recovers. Thus, some of those who develop IA despite carrying the protective AspAsp genotype may respond differently to infection than those carrying the AsnAsn genotype. An additional possibility is that carriers of the AsnAsn genotype who develop IA tend to have a more evident phenotype and consequently are either more readily diagnosed or are diagnosed at an earlier stage of disease. Thus carriers would have elevated risk of diagnosis but would present with a more clinically manageable form of IA.

Human association studies in smaller independent HSCT cohorts have implicated Toll-like receptor 1 and 6 [31] and IL-10 receptor [32] polymorphisms as risk factors for development of invasive aspergillosis, While the published human association studies target viable candidate genes based on the prior literature, they are hampered by small size (n = 22 [31] and n = 9, including 4 cases of “possible” IA [32]). Additionally, conflicting data exist on the role of other common innate immune deficiencies (i.e. TLR2 and TLR4 hypofunction) in the pathogenesis of IA [33]–[38]. Our work with C57Bl6^tlr4−/−^ mice did not find TLR4 to be essential for host defense against IA (data not shown), thus unlikely to be the sole reason for enhanced susceptibility of C3H/HeJ mice who are known to have TLR4 hypofunction. Similarly, where deficiency in complement 5a (C5a) may influence outcome in invasive aspergillosis [39], the varying phenotypes between A/J and AKR/J (both C5a deficient) mice indicate that C5a deficiency alone is unlikely to account for the poor survival of A/J mice.

A polymorphism in the gene for plasminogen may confer increased risk of invasive aspergillosis following bone marrow transplantation. The identification of murine and human genetic polymorphisms associated with susceptibility to invasive fungal infection in immunosuppressed hosts enables subsequent epidemiologic and clinical studies that can produce improved methods for management of transplant patients.

## Methods

### Immunosuppression

Inbred 6–8 week-old female mice (Jackson Laboratories; Bar Harbour, ME) were housed and fed under aseptic conditions and their sterile water was supplemented with tetracycline (1 mg/ml) changed once daily. Ten inbred strains were utilized (BalbC/ByJ, Balb/CJ, AKR/J, 129/SvJ, C57Bl/6J, MRL/MPJ, NZW/LACJ, A/J, DBA/2J and C3H/HeJ). Mice weighed between 18–24 grams, with the exception of MRL/MPJ mice (28–32 grams).

The mice were immunosuppressed with an intraperitoneal injection of cyclophosphamide (Sigma Biochemicals, St. Louis, MO) (150 mg/kg) on day −3, and a subcutaneous injection of cortisone acetate (Sigma Biochemicals, St. Louis, MO) (250 mg/kg) on day −1 of infection. The immunosuppressive regimen also included additional doses of cyclophosphamide (150 mg/kg) on days +1, and +4 of infection. All animal work was approved by the Institutional Animal Use and Care Committee at Duke University Medical Center and followed the standard guidelines for ethical treatment of animals.

### *Aspergillus fumigatus* Strain

AF strain 293 was used in all experiments (provided by Dr. William J. Steinbach, Duke University Medical Center). AF conidia were grown on Sabouraud dextrose agar (Difco; Becton Dickinson; Sparks, MD) for 7 days and harvested in 0.01% Tween 80 in sterile water on the day prior to inoculation. Conidia were washed and resuspended in sterile water and counted on a Neubauer hematocytometer to create a conidial suspension of 3×10^8^ conidia/ml.

### Inoculation with Conidia

10 mice per strain were immunosuppressed. A total of 40 ml of the 3×10^8^ conidia/ml suspension was aerosolized in four separate nebulizers (Aerotech II, CIS-US, Inc., Beford, MA) in a Hinners-style exposure chamber [40] for 25 minutes as previously described [41]. Select strains (C3H/HeJ, A/J, C57Bl/6J and Balb/CJ) were evaluated a total of 3 times each to ensure reproducibility of findings, thus the total number of mice evaluated was 160.

### Computational Haplotype Mapping

Haplotype-based computational genetic analysis of the phenotypic data was performed as previously described [4],[6],[42],[43],[44]. In brief, allelic data from multiple inbred strains were analyzed and a haplotype block map of the mouse genome was constructed. SNPs were organized into haplotype blocks. Only a limited number of haplotypes-typically 2, 3 or 4-are present within a haplotype block. This analysis identifies haplotype blocks in which the haplotypic strain grouping within a block correlates with the distribution of phenotypic data among the inbred strains analyzed. To do this, a p-value that assesses the likelihood that genetic variation within each block could underlie the observed distribution of phenotypes among the inbred strains is calculated as described using ANOVA [6],[43],[44]. The phenotypic data was evaluated using the average value for each strain, obtained by assessing 10–30 mice per strain. The haplotype blocks are then ranked based upon the calculated p-value. The genomic regions within haplotype blocks that strongly correlated with the phenotypic data are then analyzed. When the computational analysis was performed, there were 1745 haplotype blocks that were generated from analysis of 160,000 SNPs with alleles characterized across 18 inbred strains covering 2,171 genes. For this analysis, the candidate haplotype blocks that were empirically selected had p-value<0.005. This was the best p-value achieved by blocks in which the 10 strains are grouped into two haplotypes such that the phenotypes of strains in one haplotype are separated from those of strains in another haplotype.

### Mouse Genomic Sequencing

Genomic sequencing of *PLG* in inbred mouse strains was performed as previously described [4]; and covered the 2 kb 5′ upstream, 1 kb 3′ downstream, intron-exon junctions and all exonic regions.

### Quantitative *Aspergillus* PCR

AF burden was quantified in murine lung at 24 and 48 hours following inhalation of 3.0×10^8^ AF 293 conidia according to the methods of Bowman, *et al* [45]. Briefly, organ samples were mechanically disrupted using a roller-bottle method [46] and then vigorous agitation in a FastPrep 120 (Qbiogene; Carlsbad, CA) homogenizer. DNA was extracted from the homogenate using the DNeasy 96 Tissue Kit (Qiagen; Valencia, CA) according to the manufacturer's instructions. Oligonucleotide amplification primers and a dual-labeled fluorogenic oligonucleotide hybridization probe complementary to sequence from the 18S rRNA gene utilized by Bowman, et al [45]; were designed using Primer Express version 1.5 (Applied Biosystems; Foster City, CA). The sequences of these oligonucleotides are-

1. sense amplification primer: 5′- GGCCCTTAAATAGCCCGGT-3′
2. antisense amplification primer: 5′-TGAGCCGATAGTCCCCCTAA-3
3. probe: 5′-6FAM-AGCCAGCGGCCCGCAAATG-TAMRA-3′.

Modifications to the published protocol include the normalization of DNA content by a spike addition of 2×10^6^ copies of the non-murine, non-fungal plasmid *Eimeria tenella PKG* cDNA (Accession Number AF411961), with normalization of results to amount of *E. tenella* DNA extracted (Bowman, et al; unpublished data). After homogenization and DNA isolation, samples are analyzed by TaqMan with the following primers and probe specific for the parasite gene sequence:

1. sense amplification primer: 5′-AGGGCTTTGCTGCACGAC-3′
2. antisense amplification primer: 5′-TCCACCTCGGGACTGTTTG-3′
3. hybridization probe: 5′- FAM-TGCTACTGTTGCAGACCGCCGCT-TAMRA-3′

TaqMan quantification of the PKG target sequence allows for an estimate of the recovery of DNA in the experiment from the crude homogenate through the TaqMan reaction. This assessment of DNA recovery was made for each experimental sample. The percent recovery of the PKG target sequence was used to estimate the recovery of tissue DNA from the sample. Accordingly, each TaqMan data point for the AF18S rRNA gene target was normalized based on the recovery of DNA predicted by the PKG standard. Data were analyzed using GraphPad Prism version 4.0 for Windows, (GraphPad Software, San Diego, CA, www.graphpad.com).

### Fluorescent Labeling of Mouse and Human Plasminogen

Purified mouse plasminogen prepared from outbred mice (Haematologic Technologies) was dialyzed against PBS and labeled with AlexaFluor 488 per the manufacturers instructions (Invitrogen). The moles dye per mole protein binding ratio of labeled plasminogen was 5. Free dye was removed by dialyzing labeled protein for 24 hours against PBS at 4°C. Purified human glu-plasminogen (Haematologic Technologies) was labeled in the same manner, with a moles dye per mole protein binding ratio of 6.

### Plasminogen Binding to *A. fumigatus* Conidia

Swollen *A. fumigatus* conidia were prepared by incubating freshly harvested *A. fumigatus* 293 conidia in RPMI for 6 hours at 37°C. Swollen conidia were washed with PBS prior to incubation with AlexaFluor 488 labeled murine or human plasminogen. Resting and swollen *A. fumigatus* conidia (1×10^7^ conidia/ml) were treated with several concentrations (0.2 to 10 µg/ml) of AlexaFluor 488 labeled mouse or human plasminogen in PBS and incubated at 37°C for 30 minutes with shaking. As controls, swollen conidia were incubated with increasing concentrations (0.2 to 4 µg/mL) of FITC-bovine serum albumin (BSA) (Invitrogen). Plasminogen binding was quantified by flow cytometric analysis (Duke Human Vaccine Institute Flow Cytometry Core Facility and Flow Cytometric Core Facility, National Institute of Environmental Health Sciences). For competitive inhibition binding assays *A. fumigatus* conidia (1×10^7^) were first treated with unlabelled mouse or human plasminogen (Haematologic Technologies) then AlexaFluor 488 labeled mouse or human plasminogen. Controls included swollen conidia pre-incubated in PBS-0.1% BSA overnight prior to incubation with AlexaFluor 488 labeled human plasminogen as described above. Conidia were incubated at 37°C for 30 minutes with shaking for each treatment. Plasminogen binding was quantified by flow cytometric analysis (Duke Human Vaccine Institute Flow Cytometry Core Facility and Flow Cytometric Core Facility, National Institute of Environmental Health Sciences). For microscopy, conidia were treated with Calcofluor white (Sigma Biochemicals) to visualize the cell wall. Binding was visualized using a Zeiss Axioskop 2 Plus (Carl Zeiss MicroImaging) fluorescent microscope with an AxioCam MRM digital camera.

### Plasminogen Binding to *A. fumigatus* Hyphae

10 ml of RPMI-1640 medium in a T-25 vented cap tissue culture flask was inoculated with AF conidia (1×10^7^) and incubated overnight at 37°C with 5% CO_2_. Hyphae were collected by gentle vortexing of the tissue culture flask and 10 µl aliquots of hyphae were spotted onto glass slides and allowed to dry. Hyphae were then treated with AlexaFluor 488 labeled mouse or human plasminogen for 30 minutes at 37°C at varying concentrations and with Calcofluor white to visualize the cell wall. For competitive inhibition binding assays *A. fumigatus* hyphae were first treated with unlabelled mouse or human plasminogen (Haematologic Technologies) for 30 minutes at 37°C then Alexa Fluor 488 labeled mouse or human plasminogen (0.2 µg/ml) for 30 minutes at 37°C. Binding was visualized using a Zeiss Axioskop 2 Plus fluorescent microscope with an AxioCam MRM digital camera.

### Human Genomic Sequencing

Genomic DNA was prepared from patients who received allogeneic hematopoietic stem cell transplant (HSCT) after myeloablative therapy at the Fred Hutchinson Cancer Research Center (Seattle, WA), and their donors. An immortalized lymphocyte cell line was created from each subject using peripheral blood mononuclear cells obtained prior to transplantation[47]. Genomic DNA was isolated using a Qia-Amp DNA blood Kit (Qiagen). The cohort represented a sample of 83 patients who developed proven or probable invasive aspergillosis, according to standardized criteria[48], and 147 patients who did not. Cases of “possible” aspergillosis were not included in the study. The plasminogen (*PLG*) gene extending from 2 kb 5′ upstream to 1 kb 3′ downstream of human *PLG* transcript was sequenced in 20 IA affected donor/recipient pairs using Applied Biosystems (AB) Version 3.1 Dye Terminators and an ABI 3730 Genetic Analyzer (Applied Biosystems). Polymorphisms were identified, and corresponding genotypes determined, by analysis of the resulting DNA sequence data using PHRED and PHRAP. Four coding change SNPs were identified and exons containing these SNPs (exons 2, 3, 11, and 12, Ensembl Human V.36) were further sequenced for all members of the cohort to determine their genotypes using PolyPhred. To identify potentially important sequence changes in h*PLG*, all exons and the 500 bp promoter region of the h*PLG* gene were sequenced in 20 HSCT donor-recipient pairs; nineteen SNPs were identified. Of the 19 SNPs, 11 SNPs were located in exons and 4 of them induced a change in an amino acid.

Exons containing these nonsynonymous coding change SNPs (exons 2, 3, 11, and 12) were sequenced in the remaining sample of 210 Caucasian donor/recipient pairs (63 with proven/probable IA, 147 without IA) (Demographics; Table 1). SNP Asp472Asn (rs4252125) at position 827 in exon 11 was the only one of the nonsynonymous coding change SNPs considered to be of further interest as it had a minor allele frequency of approximately 25%, while the other 3 SNPs had minor allele frequencies of <1%.

Statistical Methods: A Cox proportional hazards approach was used to model the hazard of IA in relation to the recipient genotype, over follow-up time after receipt of stem cells. Death and relapse were treated as censoring mechanisms [49]. Although the required proportional hazards assumption was not significantly violated, in the final modeling the follow-up for analysis was begun on day 40 following transplantation, though this left-truncation did not materially affect the results. Because patients developing IA were sampled with probability 0.25, while those who had remained free of IA were sampled with probability 0.09, the differential sampling had to be accounted for statistically. This was accomplished by designating a random 9/25 of the IA cases (31 of 83), together with all noncases, as members of a synthetic random subcohort, and applying software for case-cohort analysis [50]. In this way, the variances for parameter estimation were increased appropriately to account for the dependency induced by using the same controls in successive risk sets in modeling the risk of IA. This weighting was also accounted for in calculating Kaplan-Meier cumulative incidence curves. For multi-category variables, the improvement in fit provided by their inclusion in a model was tested by means of chi-squared statistics based on scores. To assess the proportionality assumption required by the Cox proportional hazards model, the relative risks associated with the *PLG* genotype were permitted to vary across two defined periods of time: days 40–100 and days more than 100 following receipt of stem cells, as these time intervals represent known differential risk periods for IA [2]. Additionally, we included models that allowed genotype (and certain other factors, such as HLA matching status) to have different effects during different epochs of follow-up. Division of time into epochs was based on knowledge of how clinical approaches to management are altered in the months following HSCT. Analyses were performed both for the entire available follow-up time, and with follow-up truncated at one year. Results are shown for the analysis beginning at 40 days and truncated at one year, but the inference is not materially different with inclusion of longer follow-up. Covariates considered for adjustment as possible confounders or effect modifiers included age, cell type, malignancy category, antifungal prophylaxis, gender, genotype of the donor, and HLA status of graft [matched related (6/6 match from relative); matched unrelated (6/6 match from non-relative) or mismatched]. Adjustment for development of graft-versus-host disease was considered, but judged not to be defensible as a legitimate potential confounder of recipient genotype, based on any plausible causal directed acyclic graph [51]. Possible effects of genotype on survival following IA were explored through a Cox proportional hazards analysis, with follow-up beginning the day after diagnosis of IA. The contribution of each variable to the final model was tested by means of score tests and all p values given are two-sided.

Hardy-Weinberg equilibrium for the recipient patients was tested based on the members of the sub-cohort, while that for the donors were tested based on all the donors. Work was approved by the Institutional Review Boards of the Fred Hutchinson Cancer Research Center, Duke University Medical Center and the National Institute of Environmental Health Sciences.

### Modeling of Murine and Human Plasminogen Amino Acid Changes

A model for murine kringle-1 (K1) was created using the X-ray crystal structure of the kringle-1 domain of human plasminogen (pdb entry: 1CEA). All appropriate mutations required for this homology modeling were carried out using Sybyl 7.1 (Tripos, Inc.). The structure was then energy minimized in vacuum using the Amber force field (FF03) [52]. Gly6 was then mutated to Ser and the structure was re-minimized using the above force field. Minimization was performed using the Sander module of the molecular dynamics package Amber 9.0 [53]. All hydrogens were added to the crystal structure of human plasminogen K1 (pdb entry: 1CEA) and the sidechains were energy minimized (in vacuum) using the Amber force field (FF03). The electrostatic potential surfaces of the minimized structures were constructed using the program Grasp [54]. For modeling the change in human plasminogen, the combined K4K5 domains along with the connecting region was modeled using the crystal structures of the individual kringle domains (K4 from PDB entry 2PK4 and K5 from PDB entry 5HPG) and the X-ray crystal structure of K1-K2-K3 domains of human angiostatin. The K1-K2 relative domain arrangement in angiostatin was used as the initial template for K4 superimposed on K1 and K5 superimposed on K2 and the loop building using homology modeling package Sybyl 7.1 (Tripos, Inc.) yielded the initial structure of K4K5 domains. A subsequent minimization followed by a nanosecond molecular dynamics simulation using Generalized Born solvation model (Amber 9.0) was used to obtain the final representative structure for K4K5 of plasminogen. Asp472 in the native K4K5 structure was mutated to Asn and the resultant structure was subjected to a minimization followed by a nanosecond generalized Born salvation dynamics trajectory calculation to obtained the representative structure of the K4K5 (Asp472Asn) mutant structure.

## Supporting Information

Figure S1 (A) Computational prediction of chromosomal regions regulating outcome following immune suppression and exposure to inhaled *A. fumigatus*. Segments are arranged from centromeric to telomeric for all 19 autosomes. Each bar represents a 30-cM interval, and neighboring bars are offset by 10 cM. The dotted line represents a useful cutoff for analyzing this data; the most highly correlated 10% of the loci are above this line. (B) After exclusion of the DBA/2J strain, the interval containing Plg remains among the top predicted loci.
(0.03 MB PDF)

Figure S2 Computational genetic analysis identifies plasminogen (Plg) as a candidate susceptibility gene for invasive aspergillosis. Haplotype-based computational genetic analysis was used to analyze the area under the survival curve to identify genetic factors affecting survival after AF exposure among the inbred strains. The area under the survival curve (AUC) after AF exposure was analyzed for 9 inbred strains. There were several haplotype blocks that had a strong correlation with survival curve AUC with P value less than 0.01, including the block containing Plg. However, Plg remained a top biologically plausible candidate. Red blocks indicate haplotypes for the “resistant” strains (Balb/CJ, Balb/CbyJ, C57Bl/6J, AKR/J and 129/SvJ) and the “intermediate” strain NZW/LacJ, and the blue blocks indiciate “susceptible” strains (C3H/HeJ and A/J) and the “intermediate” strain MRL/MpJ.
(0.19 MB PDF)

Figure S3 Ribbon diagrams of (A) human (C) murine (E) murine (Gly6Ser) plasminogen kringle-1 structures. Amino acid numbering for this model is used for kringle 1 only, thus Gly6 in this model corresponds to Gly110 in the full plasminogen structure. Gly6 (and Ser6 in the mutated structure) are shown as space filling models on the ribbon diagram. Also shown are Asp55 and Asp57 (above Gly6), the key lysine binding residues in kringle-1. Corresponding electrostatic potential surfaces are shown in (B) for human K-1, (D) for murine, and (F) for murine (Gly6Ser) structures. Notably, the negativity of the electrostatic potential surface is enhanced in the variant plasminogen, thus possibly increasing the affinity of lysine binding. The area of the lysine binding site is circled.
(0.48 MB PDF)

Table S1 White blood cell (WBC) and absolute neutrophil counts (ANC) were measured in each mouse strain at the indicated times after administration of cyclophosphamide and cortisone acetate (n = 5 mice/strain). Neutropenia/leukopenia are present at time of inhalation (day 0) through day 7, and recover by day 10 following inhalation. The pulmonary Aspergillus burden was measured by RT-PCR 24 and 48 hours after inhalation of 3.0×10e8 AF conidia. Of note, all mice had equivalent fungal burden 24 hours after AF exposure (P = NS, Wilcoxon rank sum). However, 48 hours after AF exposure, mice from sensitive inbred strains (A/J and C3H/HEJ, n = 4 mice/strain) had increased fungal burden from initial values (median conidial equivalents 4.7 log cells/gram lung tissue; range 3.8–5.0) versus resistant inbred strains (AKR/J, 129/SvJ, C57/Bl6, Balb/C and Balb/CByJ; n = 4–5 mice/strain) did not increase their fungal burden (median conidial equivalents 3.7 log cells/gram lung tissue; range 2.7–5.3; p = 0.02, Mann-Whitney test).
(0.10 MB DOC)
