# Supplementary figures and images for: Plasminogen Alleles Influence Susceptibility to Invasive Aspergillosis
Source: PLoS Genet. 2008 Jun 20;4(6):e1000101. doi: 10.1371/journal.pgen.1000101 (PMC2423485; doi:10.1371/journal.pgen.1000101)

Supplementary Figure 1A

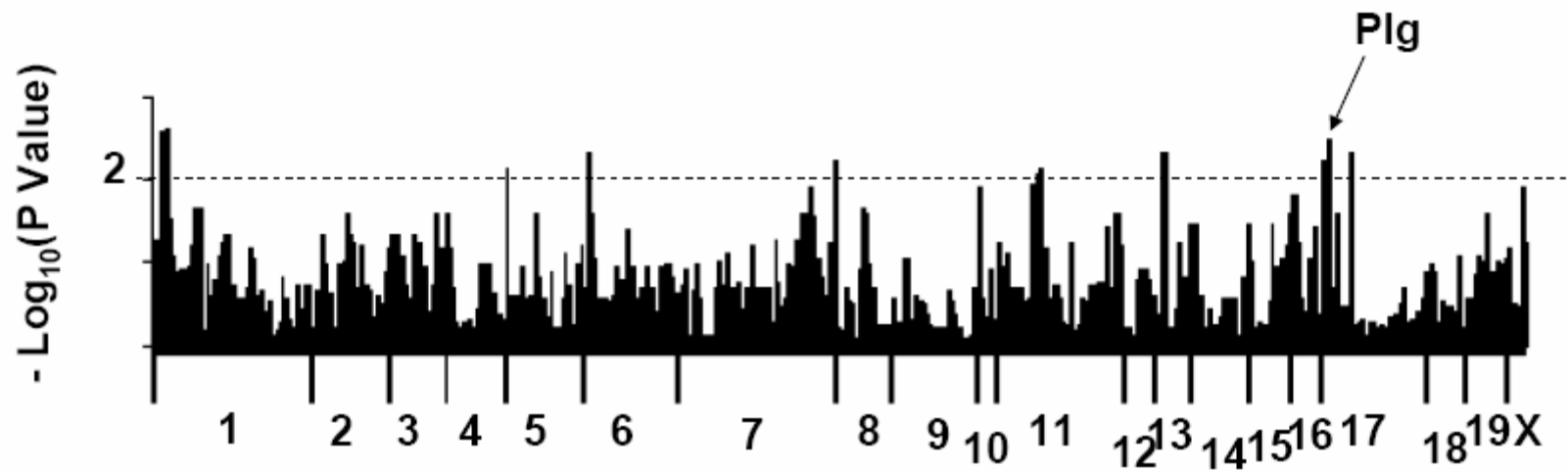

Supplementary Figure 1B

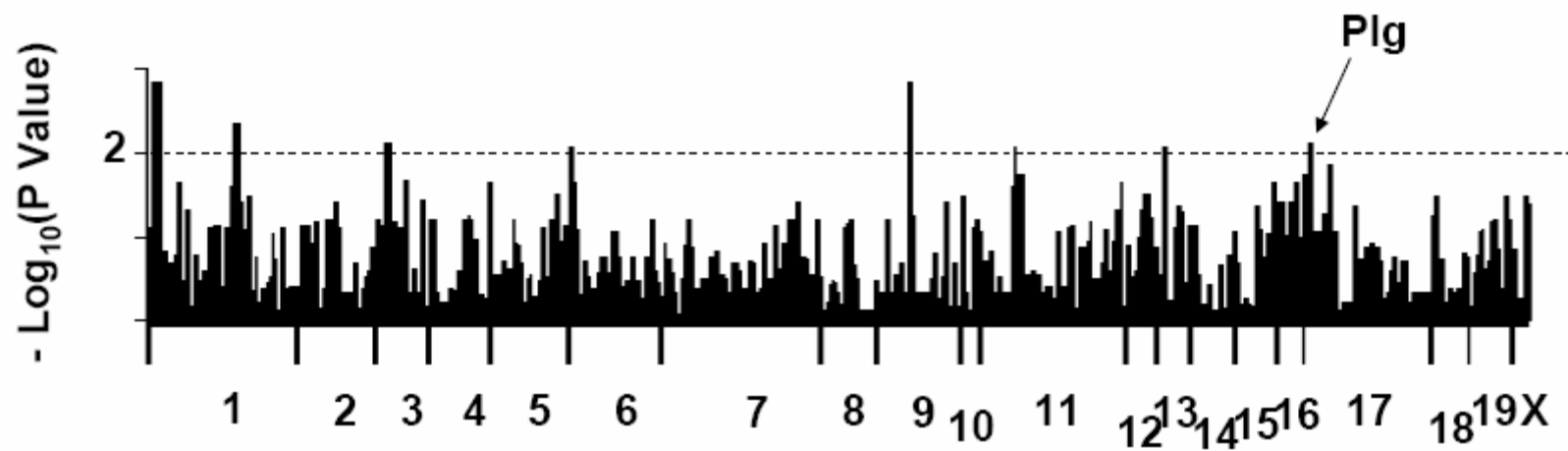

Supplement: Figure S1 — (A) Computational prediction of chromosomal regions regulating outcome following immune suppression and exposure to inhaled A. fumigatus. Segments are arranged from centromeric to telomeric for all 19 autosomes. Each bar represents a 30-cM interval, and neighboring bars are offset by 10 cM. The dotted line represents a useful cutoff for analyzing this data; the most highly correlated 10% of the loci are above this line. (B) After exclusion of the DBA/2J strain, the interval containing Plg remains among the top predicted loci. (0.03 MB PDF) [file pgen.1000101.s001.pdf]

### Supplementary Figure 3:

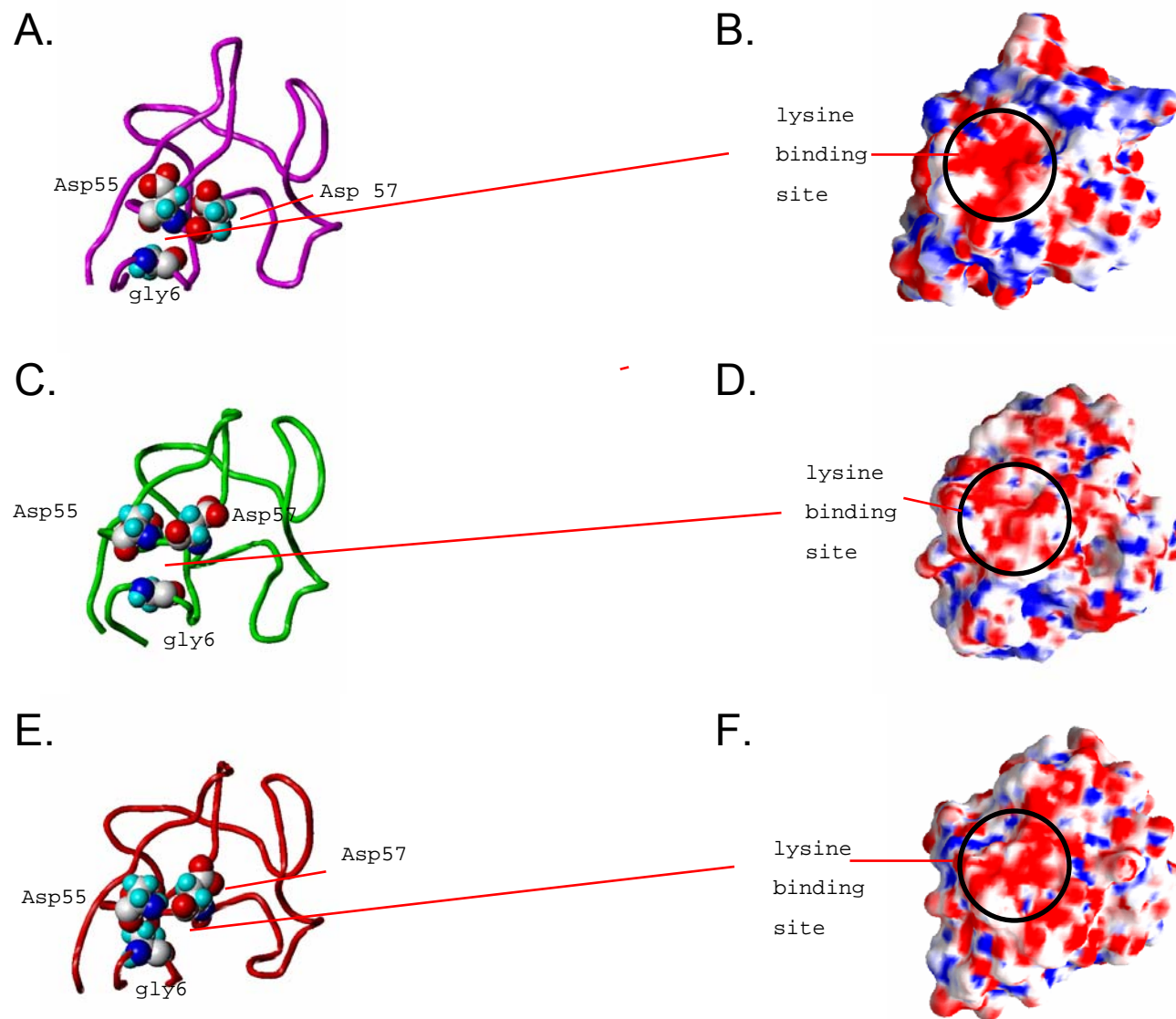

Supplement: Figure S3 — Ribbon diagrams of (A) human (C) murine (E) murine (Gly6Ser) plasminogen kringle-1 structures. Amino acid numbering for this model is used for kringle 1 only, thus Gly6 in this model corresponds to Gly110 in the full plasminogen structure. Gly6 (and Ser6 in the mutated structure) are shown as space filling models on the ribbon diagram. Also shown are Asp55 and Asp57 (above Gly6), the key lysine binding residues in kringle-1. Corresponding electrostatic potential surfaces are shown in (B) for human K-1, (D) for murine, and (F) for murine (Gly6Ser) structures. Notably, the negativity of the electrostatic potential surface is enhanced in the variant plasminogen, thus possibly increasing the affinity of lysine binding. The area of the lysine binding site is circled. (0.48 MB PDF) [file pgen.1000101.s003.pdf]
