# Supplementary material for: Plasminogen Alleles Influence Susceptibility to Invasive Aspergillosis
Source: PLoS Genet. 2008 Jun 20;4(6):e1000101. doi: 10.1371/journal.pgen.1000101 (PMC2423485; doi:10.1371/journal.pgen.1000101)
Supplement: Figure S2 — Computational genetic analysis identifies plasminogen (Plg) as a candidate susceptibility gene for invasive aspergillosis. Haplotype-based computational genetic analysis was used to analyze the area under the survival curve to identify genetic factors affecting survival after AF exposure among the inbred strains. The area under the survival curve (AUC) after AF exposure was analyzed for 9 inbred strains. There were several haplotype blocks that had a strong correlation with survival curve AUC with P value less than 0.01, including the block containing Plg. However, Plg remained a top biologically plausible candidate. Red blocks indicate haplotypes for the “resistant” strains (Balb/CJ, Balb/CbyJ, C57Bl/6J, AKR/J and 129/SvJ) and the “intermediate” strain NZW/LacJ, and the blue blocks indiciate “susceptible” strains (C3H/HeJ and A/J) and the “intermediate” strain MRL/MpJ. (0.19 MB PDF) [file pgen.1000101.s002.pdf]

Supplementary Figure 2

P-value	Haplotype	Chr.	Position (MB)	#SNPs	Symbol
0.0015	🟦🟦🟦🟦🟥🟥🟥🟥🟥	1	36.43~37.55	86	Ugcgl1
0.0015	🟦🟦🟦🟦🟥🟥🟥🟥🟥	1	37.55~37.55	8	Cnga3
0.0015	🟦🟦🟦🟦🟥🟥🟥🟥🟥	9	58.12~58.13	12	Cyp11a1
0.0048	🟥🟥🟥🟥🟥🟦🟦🟦🟦	1	170.89~170.90	20	Fcgr2b
0.008	🟥🟥🟥🟥🟦🟥🟦🟦🟦	3	64.97~64.97	23	Kcnab1
0.008	🟥🟥🟥🟥🟦🟥🟦🟦🟦	17	11.05~11.30	26	Plg
0.008	🟥🟥🟥🟥🟦🟥🟦🟦🟦	17	11.30~11.30	4	Slc22a1
0.0089	🟥🟥🟥🟥🟥🟥🟦🟦🟦	6	50.48~50.49	10	Osbpl3
0.0089	🟥🟥🟥🟥🟥🟥🟦🟦🟦	11	56.94~56.95	4	Gria1
0.0089	🟥🟥🟥🟥🟥🟥🟦🟦🟦	13	57.44~57.44	10	nb:Ntrk2
