# Supplementary material for: Plasminogen Alleles Influence Susceptibility to Invasive Aspergillosis
Source: PLoS Genet. 2008 Jun 20;4(6):e1000101. doi: 10.1371/journal.pgen.1000101 (PMC2423485; doi:10.1371/journal.pgen.1000101)
Supplement: Table S1 — White blood cell (WBC) and absolute neutrophil counts (ANC) were measured in each mouse strain at the indicated times after administration of cyclophosphamide and cortisone acetate (n = 5 mice/strain). Neutropenia/leukopenia are present at time of inhalation (day 0) through day 7, and recover by day 10 following inhalation. The pulmonary Aspergillus burden was measured by RT-PCR 24 and 48 hours after inhalation of 3.0×10e8 AF conidia. Of note, all mice had equivalent fungal burden 24 hours after AF exposure (P = NS, Wilcoxon rank sum). However, 48 hours after AF exposure, mice from sensitive inbred strains (A/J and C3H/HEJ, n = 4 mice/strain) had increased fungal burden from initial values (median conidial equivalents 4.7 log cells/gram lung tissue; range 3.8–5.0) versus resistant inbred strains (AKR/J, 129/SvJ, C57/Bl6, Balb/C and Balb/CByJ; n = 4–5 mice/strain) did not increase their fungal burden (median conidial equivalents 3.7 log cells/gram lung tissue; range 2.7–5.3; p = 0.02, Mann-Whitney test). (0.10 MB DOC) [file pgen.1000101.s004.doc]

| **Supplemental Table 1** |  |  |  |  |  |  |  |  |  |
| --- | --- | --- | --- | --- | --- | --- | --- | --- | --- |
| **Strain** | **Day 0** |  |  |  | **Day 7** |  |  |  |  |
|  | **WBC (cells/uL)** |  | **ANC** |  | **WBC (cells/uL)** |  | **ANC** |  |  |
|  |  | **StdDev** |  | **StdDev** |  | **StdDev** |  | **StdDev** |  |
| C3H/HeJ | 0.533 | 0.168 | 0.288 | 0.146 | 0.108 | 0.062 | 0.008 | 0.011 |  |
| A/J | 0.237 | 0.125 | 0.074 | 0.093 | 0.212 | 0.53 | 0.065 | 0.13 |  |
| NZW/LACJ | 0.848 | 0.458 | 0.138 | 0.061 | 0.161 | 0.096 | 0.003 | 0.002 |  |
| MRL/MPJ | 0.522 | 0.173 | 0.218 | 0.106 | 0.341 | 0.414 | 0.04 | 0.041 |  |
| 129/SvJ | 0.264 | 0.091 | 0.096 | 0.050 | 0.242 | 0.238 | 0.006 | 0.002 |  |
| AKR/J | 0.646 | 0.120 | 0.393 | 0.045 | 0.164 | 0.137 | 0.021 | 0.031 |  |
| C57Bl/6J | 0.184 | 0.079 | 0.036 | 0.009 | 0.173 | 0.047 | 0.066 | 0.029 |  |
| BalbC/J | 0.968 | 0.325 | 0.452 | 0.325 | 0.391 | 0.192 | 0 | 0 |  |
| BalbC/ByJ | 0.788 | 0.143 | 0.212 | 0.061 | 0.699 | 0.439 | 0.106 | 0.082 |  |
|  | **Day 10** |  |  |  | **Day 14** |  |  |  |  |
|  | **WBC (cells/uL)** |  | **ANC** |  | **WBC (cells/uL)** |  | **ANC** |  |  |
|  |  | **StdDev** |  | **StdDev** |  | **StdDev** |  | **StdDev** |  |
| C3H/HeJ | 3.499 | 0.700 | 2.061 | 0.387 | 9.926 | 1.887 | 7.888 | 3.101 |  |
| A/J | 7.237 | 3.473 | 5.757 | 3.092 | 5.956 | 2.360 | 3.667 | 1.820 |  |
| NZW/LACJ | 6.187 | 2.562 | 1.908 | 3.094 | 8.136 | 3.200 | 8.113 | 2.194 |  |
| MRL/MPJ | 4.656 | 2.302 | 3.199 | 1.989 | 4.876 | 2.751 | 3.178 | 2.228 |  |
| 129/SvJ | 3.51 | 1.021 | 1.758 | 0.510 | 7.586 | 1.101 | 5.197 | 1.432 |  |
| AKR/J | 4.32 | 3.590 | 2.873 | 3.208 | 3.694 | 0.836 | 2.67 | 0.680 |  |
| C57Bl/6J | 7.338 | 2.081 | 6.206 | 2.228 | 3.768 | 3.906 | 2.082 | 1.732 |  |
| BalbC/J | 3.888 | 0.859 | 1.307 | 0.266 | 6.45 | 1.637 | 5.34 | 1.49 |  |
| BalbC/ByJ | 7.415 | 4.29 | 4.396 | 1.644 | 6.092 | 2.799 | 5.82 | 2.736 |  |
|  |  |  |  |  |  |  |  |  |  |
|  | **24 hour Aspergillus Burden** |  |  |  |  |  |  |  |  |
|  |  |  | **48 hour Aspergillus Burden** |  |  |  |  |  |  |
|  | **(logCE/gramlung)** |  | **(logCE/gramlung)** |  |  |  |  |  |  |
|  | **Mean** |  | **StdDev** |  | **Mean** |  | **StdDev** |  |  |
| C3H/HeJ | 3.44 |  | 0.55 |  | 4.79 |  | 0.17 |  |  |
| A/J | 2.98 |  | 0.48 |  | 4.43 |  | 0.64 |  |  |
| NZW/LACJ | 3.06 |  | 0.42 |  | 3.47 |  | 0.32 |  |  |
| MRL/MPJ | 2.95 |  | 0.47 |  | 3.31 |  | 0.37 |  |  |
| 129/SvJ | 3.31 |  | 0.2 |  | 3.76 |  | 0.28 |  |  |
| AKR/J | 2.92 |  | 0.27 |  | 4.7 |  | 0.35 |  |  |
| C57Bl/6J | 3.07 |  | 0.19 |  | 3.36 |  | 0.33 |  |  |
| BalbC/J | 3.19 |  | 0.5 |  | 3.85 |  | 0.79 |  |  |

Supplementary Table 1: White blood cell (WBC) and absolute neutrophil counts (ANC) were measured in each mouse strain at indicated times after administration of cyclophosphamide and cortisone acetate (n=5 mice/strain). Neutropenia and leucopenia are present at time of inhalation (day 0) through day 7 and recover by day 10 following inhalation. The pulmonary *Aspergillus* burden was measured by RT-PCR 24 and 48 hours following inhalation of 3.0x10^8^ conidia/mL in 40 mL sterile PBS. Of note, all strains had equivalent fungal burden at 24 hours post inhalation (P=NS, Wilcoxon rank sum).

However, 48 hours after inhalation, the sensitive strains (A/J, C3H/HeJ, n =4 mice per strain) had increased fungal burden (median conidial equivalents 4.7 log cells/gram tissue, range 3.8-5.0) versus resistant strains (AKR/J, C57Bl6/J, 129/SvJ, Balb.CJ and Balb/CByJ, n=4-5 mice per strain) who did not increase fungal burden (median conidial equivalents 3.7 log cells/gram tissue, range 2.7-5.3, p = 0.02, Mann-Whitney U Test).
